# Outcomes associated with the use of a new powered circular stapler for left-sided colorectal reconstructions: a propensity score matching-adjusted indirect comparison with manual circular staplers

**DOI:** 10.1007/s00464-021-08542-7

**Published:** 2021-05-24

**Authors:** Patricia Sylla, Peter Sagar, Stephen S. Johnston, Harikumaran R. Dwarakanathan, Jason R. Waggoner, Michael Schwiers, Sanjoy Roy

**Affiliations:** 1 Division of Colon and Rectal Surgery, Icahn School of Medicine at Mount Sinai, New York, NY USA; 2 John Goligher Colorectal Department, St James’ University Hospital, Leeds, UK; 3 Real-World Data Analytics and Research, Epidemiology, Medical Devices, Johnson & Johnson, 410 George Street, New Brunswick, NJ USA; 4 MuSigma, Bengaluru, Karnataka India; 5 Ethicon Endo-Surgery, Inc, Cincinnati, OH USA

**Keywords:** Circular stapler, Complications, Readmissions, Colorectal resection

## Abstract

**Background:**

This was a retrospective, matching-adjusted indirect comparison of clinical outcomes between patients from a single-arm trial of the ECHELON CIRCULAR™ Powered Stapler (ECP) and those from a historical cohort of patients who underwent left-sided colorectal resection using conventional manual circular staplers, extracted from the Premier Healthcare Database.

**Methods:**

Patients in the ECP trial cohort were propensity score matched to those in the historical cohort through nearest neighbor matching. Outcomes included 30-day readmission rates; length of stay (LOS) for the index admission; rates of anastomotic leak, pelvic abscess, ileus/small bowel obstruction, infection, bleeding, and stoma creation.

**Results:**

The study included 168 patients in the ECP trial cohort and 4544 patients in the historical cohort; 165 ECP trial patients were matched to 1348 historical cohort patients. After matching, conversions were more prevalent in the historical cohort than the ECP trial cohort (4.2% ECP vs. 10.2% historical, *p* = 0.001). Relative to the historical cohort, the ECP trial cohort had statistically significant lower rates of 30-day inpatient readmission (6.1% vs. 10.8%, *p* = 0.019), anastomotic leak (1.8% vs. 6.9%, *p* < 0.001), ileus/small bowel obstruction (4.8% vs. 14.7%, *p* < 0.001), infection (1.8% vs. 5.7%, *p* = 0.001), and bleeding (1.8% vs. 9.2%, *p* < 0.001) during the index admission or within 30 days thereafter. No statistically significant differences in rates of pelvic abscess, stoma creation, or LOS were found between the two cohorts. Three sensitivity analyses to address the difference in conversion rates yielded largely consistent results, with loss of statistical significance for inpatient admission in some cases. This study is limited by its potential for differences in unmeasurable factors between the ECP trial and historical cohorts.

**Conclusions:**

In this study, the ECP trial cohort had lower incidence proportions of several surgical complications as compared with the historical cohort. Further controlled prospective clinical studies are needed to confirm the validity of this finding.

**Supplementary Information:**

The online version contains supplementary material available at 10.1007/s00464-021-08542-7.

Circular staplers are widely used to create circular anastomoses during colorectal reconstructions. Until recently, all circular staplers were fired under manual grip force. While circular staplers typically provide reliable tissue apposition during firing, there is potential for suboptimal tissue tension from variable operator workload and straining during stapling based on individual grip strength. Manual circular staplers have been associated with technical errors and low satisfaction – particularly among surgeons with smaller glove sizes [1, 2].

In March of 2019, the first powered circular stapler, the ECHELON CIRCULAR™ Powered Stapler (ECP, Ethicon Endo-Surgery, Inc., Cincinnati, OH, USA) was launched in the US and European markets. Powered stapling systems are operated using battery packs, thereby compensating for the variable grip force of the surgeon and potentially reducing movement at the distal tip of the stapler to allow for more stable stapler positioning and staple line formation.

An ex vivo preclinical model confirmed that relative to manual circular staplers, ECP required less force to fire, was associated with less movement during stapler placement, and demonstrated a higher leak (pressure) resistance [3]. A second analysis reported satisfactory safety results and anastomotic integrity among 17 left-sided anastomoses performed with ECP [4].

A recently published retrospective, single-center study by Pla-Marti and colleagues [5] compared the risk of anastomotic leak after left-sided colorectal anastomosis when using the ECP (61 patients) versus manual circular staplers (218 patients). They reported anastomotic leak in 11.8% of patients in the manual circular stapler group and 1.7% of patients in the ECP group (*p* = 0.022). To our knowledge, however, no other studies have compared outcomes between the ECP and manual circular staplers.

A single-arm post-market multicenter trial to assess the intraoperative performance of ECP during left-sided colectomy procedures was also recently completed [6]. In this trial, ECP exhibited few technical issues, a favorable safety profile, and ease of use for creation of left-sided anastomoses as reported by operating surgeons. However, due to the single-arm nature of the ECP trial, no comparisons were made with conventional manual circular staplers.

Therefore, building upon the current state of evidence regarding the ECP, we conducted a retrospective comparison of clinical outcomes between patients from the single-arm ECP trial and those from a matched historical cohort of patients from the Premier Healthcare Database (PHD) who underwent left-sided colorectal resection using conventional manual circular staplers.

## Materials and methods

### Indirect comparison framework

This study used the framework of matching-adjusted indirect comparison, known as MAIC, to compare the outcomes of ECP trial patients to those of a historical cohort [7]. MAIC is a widely used technique to compare outcomes between independently conducted studies, and is most often implemented when individual patient data (IPD) are available for only one of the two studies under comparison (i.e., investigators have access to IPD only for their own trial and wish to compare the outcomes of their own trial relative to a previously published trial). In such a case, a form of weighting is applied to the IPD in order to reweight the IPD trial population to be reflective of the previously published trial population. In the present study, however, IPD were available for both the ECP trial and the historical cohort, allowing for the use of (a) propensity score matching to directly balance the two cohorts on important prognostic characteristics and (b) traditional statistical significance testing for inference.

### Data sources and patients

#### ECP trial cohort

Complete details on the procedures for the ECP trial (ClinicalTrials.gov registry number NCT03326895) are described by Herzig et al. [6]. Briefly, the ECP trial was a prospective, single-arm trial that enrolled 168 consecutive patients from 12 sites (6 in the US and 6 in Europe) from April 10, 2019 through January 15, 2020. Sites entered the study at different times throughout this period. Within a site, consecutive subjects who met all eligibility criteria were approached for participation in the study. Eligible patients underwent elective left-sided colorectal resections with anastomoses performed with the 29 mm or 31 mm ECP staplers. Eligible patients were also required to provide consent and express willingness to comply with all study-related evaluations and be aged 18 years or older; additional specific selection criteria are described in Herzig et al. [6].

Each of the 12 site/investigator’s Institutional Review Board (IRB) or Independent Ethics Committee (IEC) approved the protocol and consent form. Informed consent was obtained for all patients. The study was conducted in accordance with Good Clinical Practice (GCP) and the Declaration of Helsinki, as well as any other applicable local, state and federal requirements.

#### Historical cohort

The historical cohort was extracted from the Premier Healthcare Database® (PHD), a population-based hospital discharge database that contains administrative records from over 700 US hospitals that are members of the Premier healthcare performance improvement alliance, representing approximately 25% of annual US inpatient discharges [8]. PHD includes discharge-level information on patient demographics, diagnoses, procedures, medical supplies, costs, and hospital and provider characteristics. The PHD has been widely used for observational medical research, forming the basis of over 600 peer-reviewed publications since 2006.

Criteria for inclusion of PHD patients in the study’s historical cohort included elective inpatient admission with an ICD-10-PCS code for left hemicolectomy, low anterior resection (LAR), or sigmoidectomy as a primary procedure performed between October 1, 2016 and December 31, 2018 (prior to the early 2019 US launch of ECP). The first inpatient admission meeting these criteria was defined as the index admission.

Patients were identified as having undergone stapled colorectal anastomosis with a circular stapler used during the index admission based on records in each hospital’s charge master, which is a comprehensive administrative record of billable procedures, equipment fees, supplies, devices, and room and board, among other items. These records were searched for various combinations of model numbers and names of manual circular staplers. The search strategy was initially developed by two members of our research team and resultant descriptors were independently evaluated for accuracy by a third research team member. Patients who were transferred from another institution were excluded from the historical cohort. Finally, since patients in the ECP trial were all enrolled in urban hospitals with 400 beds or more, PHD patients with index admissions in rural hospitals and/or in hospitals with 399 or fewer beds were also excluded from the historical cohort.

This analysis of the PHD was conducted under an exemption from Institutional Review Board oversight for US-based studies using de-identified healthcare records, as dictated by Title 45 Code of Federal Regulations (45 CFR 46.101(b)(4)) [9].

### Outcome measures

The set of outcome measures from the ECP trial was evaluated to identify those that could also be ascertained from the healthcare records contained within the PHD. For specific surgical complications, the complete set of adverse event terms recorded for the ECP trial was evaluated to identify those that were potentially clinically relevant (e.g., whereas ‘anastomotic leak’ was included for potential analysis, ‘insomnia’ was not). The following outcomes were identified as being available in both data sources: conversion from a minimally invasive approach to open surgery; 30-day readmission rates; length of stay for the index admission; rates of: anastomotic leak; pelvic abscess; ileus/small bowel obstruction; infection (including surgical site infection [SSI], sepsis, and peritonitis); bleeding (diagnoses related to hemorrhagic complications); and ostomy creation. Supplemental Appendix 1 includes a detailed listing of the ECP trial adverse event terms along with the corresponding International Classification of Diseases, 10th Revision, Clinical Modification and Procedure Classification System (ICD-10-CM/ICD-10-PCS) diagnosis and procedure codes for the complications of interest that were observed in the historical cohort. As there is no specific diagnosis code for anastomotic leak in the ICD-10-CM taxonomy, anastomotic leak surrogate diagnoses were used following the coding conventions of Kang et al. (2013) based on ICD-9-CM to ICD-10-CM forward mapping and omitting code K91.3: post-procedural intestinal obstruction [10]. Furthermore, the ICD-10-CM taxonomy only began to delineate between superficial, deep incisional, organ and space SSIs as of October 2018, prior to which all such SSIs were coded to the same codes. Thus, the present definition of SSI included all potential forms of SSI severe enough to warrant clinical documentation in a patient’s healthcare record (see Supplemental Appendix 1).

In the ECP trial, these outcomes were collected across multiple visits including the surgery and postoperative period through discharge and the postoperative visit at 28 ± 14 days. In addition, if any unscheduled visits were completed, outcome data was also captured during these additional visits. Outcomes for the historical cohort were measured using ICD-10-CM/PCS codes from the index admission, or during inpatient, outpatient, or emergency room visits occurring within 30 days of index admission at the same hospital; presentation of patients to other hospitals could not be tracked.

For the historical cohort, two measures of 30-day readmission were created, one based only on any all-cause inpatient readmission (regardless of admission to a specific ward) and another that comprised a composite of either all-cause inpatient readmission, emergency room visit, or unscheduled outpatient visit occurring at the same hospital as the index admission; the latter definition is more aligned with the ECP trial definition of readmission whereas the former is more conservative in favor of the historical cohort.

### Matching covariates

The set of baseline patient and hospital characteristics available from the ECP trial were evaluated to identify those that may also be ascertained from the healthcare records contained within the PHD. The following baseline patient and hospital characteristics were identified as being available in both data sources: age, sex, Hispanic ethnicity, insurance type (Medicare vs. other), diabetes, hypertension, surgical approach (open, laparoscopic, or robotic), indication for surgery (colorectal carcinoma, colorectal polyps or polyposis syndrome, diverticulitis, inflammatory bowel disease, or other), teaching vs. non-teaching hospital, and hospital bed size category (400–499 vs. 500 +). As noted above, all hospitals were located in an urban setting.

### Statistical analyses

Patients in the ECP trial cohort were propensity score matched to those in the historical cohort using nearest neighbor matching with a caliper of 0.20, which was determined to be the optimal caliper after testing calipers of 0.10 and 0.15. The propensity score match accounted for all covariates listed in the Matching Covariates section above. A goal common to all matched observational studies is to maximize sample size while minimizing confounding through covariate balance after matching. Given the larger sample size of the pre-matching historical cohort (4,544 patients), ECP trial patients were matched to the historical cohort with a target ratio of 1:10; thus, depending on the number historical cohort patients with propensity scores falling within the caliper of a given ECP trial patient, an ECP trial patient could be matched with anywhere from a minimum of 1 to a maximum of 10 historical cohort patient (i.e., 1:n variable ratio matching). When feasible and not detrimental to balance, as was the case of the present study, using 1:n variable-ratio matching has been demonstrated to increase precision in cohort studies [11]. Attainment of post-match balance for matching covariates between the study arms was verified using absolute standardized mean differences (SMD); SMDs with a value < 0.10 are indicative of good balance [12].

After propensity score matching, univariable generalized linear models were used to test for statistically significant differences in the incidence proportion of outcomes between the study cohorts and generate mean incremental differences (conditional marginal effects) with 95% confidence intervals [13]. The models used a logit link and binomial error distribution for binary outcomes and a log link and negative binomial distribution for length of stay. A *p* value of < 0.05 was set a priori as the threshold for statistical significance. Statistical analyses were performed in StataSE 16 (StataCorp, College Station, Texas, US).

### US sub-analysis

Because practice and reimbursement patterns related to the duration of hospitalization differ substantially between the US and other countries, analyses of LOS were restricted to patients from the ECP trial who were enrolled in US sites. Furthermore, to evaluate the impact of geographic location (US vs Europe) on all other clinical outcomes, propensity score matching of US ECP trial patients and the historical cohort was conducted in a sub-analysis of the US cohort.

### Post-hoc sensitivity analysis involving conversions

As shown in the Results below, the proportion of patients undergoing conversion from minimally invasive to open surgery was lower for the ECP trial cohort than the historical cohort (4.2% vs. 10.2%, *p* = 0.001). As surgical conversions are often undertaken prior to performing the circular anastomosis, the absence of this event may be a surrogate for expertise with minimally invasive procedures, experience of surgeons, and differences in the difficulties of procedures. Therefore, several post hoc sensitivity analysis were conducted. First, an analysis was conducted wherein all study outcomes were reanalyzed when adjusting for the difference in conversions between the ECP trial cohort and the historical cohort. Second, an analysis was conducted when excluding patients who were converted to open surgery from the historical cohort but not excluding them from the ECP cohort (i.e. conservatively allowing converted patients to remain in the ECP cohort but not in the historical cohort); in this analysis a further conservative step was taken to include cluster-robust standard errors at the institution level, which reduces the risk of type 1 error. Finally, an analysis was conducted when stratifying the historical cohort into tertiles with respect to their institution-level conversion rate based on the patients included in the historical cohort, again using cluster-robust standard errors. For example, if a patient was operated on in an institution for which a total of 60 patients were in the historical cohort, 6 of whom were converted, their institution-level conversion rate would be 10% (6/60); if a patient was operated on in an institution for which a total of 60 patients were in the historical cohort, 1 of whom was converted, their institution-level conversion rate would be 1.7% (1/60). In this third sensitivity analysis, the ECP cohort remained pooled, allowing comparison between historical cohort patients in the bottom tertile (average 1.5% conversion rate) to the ECP cohort overall (4.2% overall conversion rate) separately from the historical cohort patients in the middle (average 10.6% conversion rate) and top tertiles (average 19.7% conversion rate). The second and third sensitivity analyses were applied only to the overall analysis sample.

## Results

### Overall analyses

**Fig. 1:**
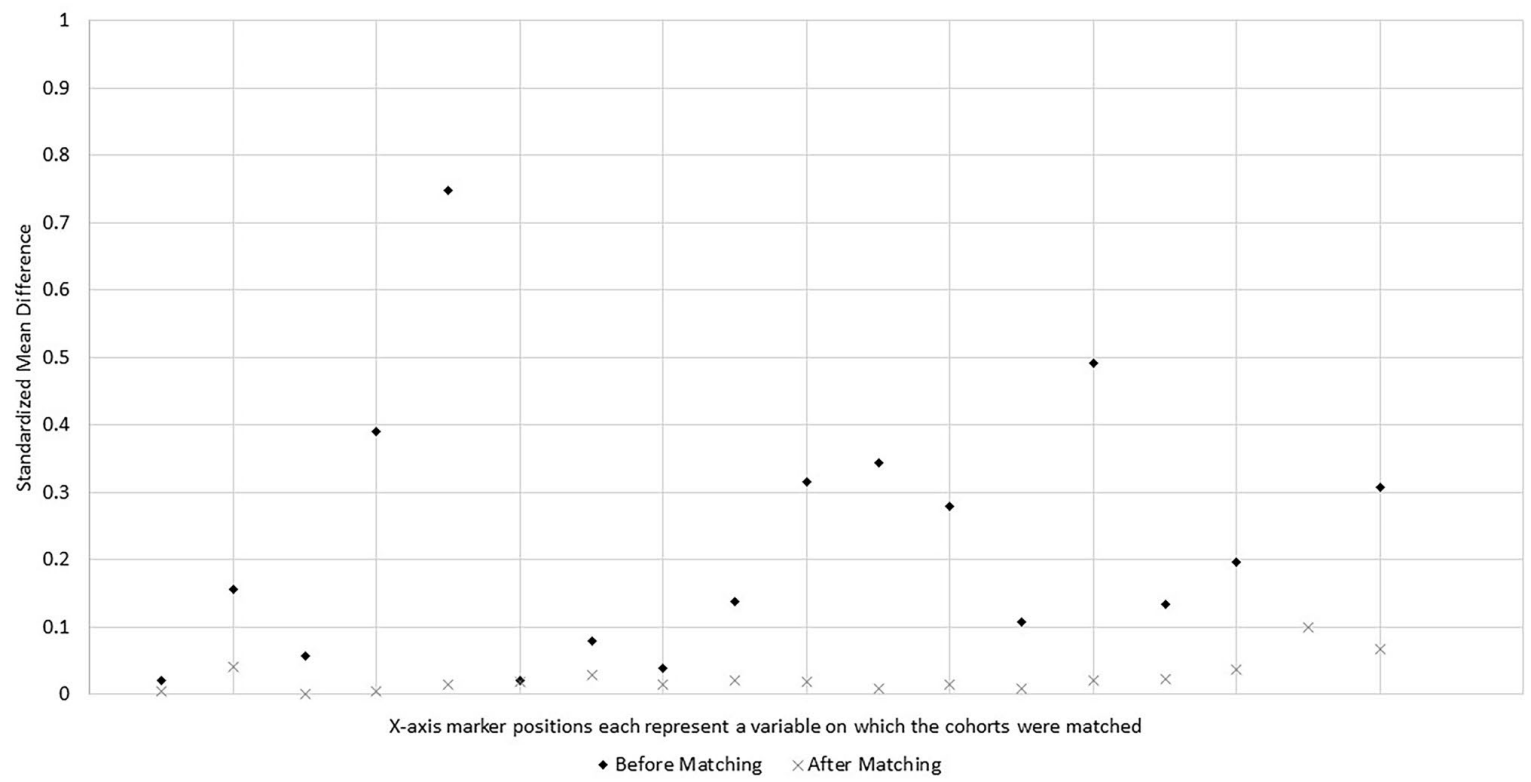
Primary analysis: Standardized mean differences before and after matching*. *A standardized mean difference <0.10 is considered indicative of good covariate balance; one standardized mean difference value before matching was 2.34 and is not shown for visual scaling purposes

Baseline characteristics of the study cohorts before and after propensity score matching are shown in Table 1. A total of 4,544 patients met the eligibility criteria for inclusion in the historical cohort and there were 168 patients in the ECP trial cohort. Before propensity score matching, the two cohorts were imbalanced (as indicated by an SMD value ≥ 0.10) with respect to several baseline characteristics (Fig. 1). Relative to the historical cohort, ECP trial patients had a lower proportion of females (47.3% vs. 55.1%) and a higher proportion of non-Hispanic or Latino patients (89.9% vs. 78.9%). Furthermore, a higher proportion of patients in the ECP trial cohort underwent surgery via the robotic approach (45.8% vs. 28.4%), surgery for colorectal carcinoma (44.6% vs. 31.0%) rather than for diverticulitis (31.5% vs. 53.9%). ECP trial sites were predominantly teaching hospitals (96.4% vs. 57.1%), and hospitals with 500 or more beds (39.3% vs. 23.9%).

**Table 1:** Primary analysis: Cohort characteristics before and after propensity score matching

	Before propensity score matching					After propensity score matching				
	Historical Cohort		ECP trial cohort		SMD	Historical Cohort		ECP trial cohort		SMD
N	4544	100.0%	168	100.0%		1348	100.0%	165	100.0%	
Age, mean / SD	59.7	12.8	59.9	13.0	0.021	60.2	13.6	60.2	12.8	0.004
Female, n / %	2504	55.1%	79	47.3%	0.156	617	45.8%	79	47.9%	0.042
Hispanic ethnicity, n / %										
Hispanic	424	9.3%	13	7.7%	0.058	107	7.9%	13	7.9%	0.001
Not Hispanic or Latino	3587	78.9%	151	89.9%	0.39	1,219	90.5%	149	90.3%	0.005
Not reported	533	11.7%	4	2.4%	0.748	22	1.6%	3	1.8%	0.014
Diabetes*, n / %	715	15.7%	25	14.9%	0.021	214	15.9%	25	15.2%	0.02
Hypertension*, n / %	2220	48.9%	75	44.6%	0.079	593	44.0%	75	45.5%	0.029
Medicare insurance (vs. other)	1708	37.6%	66	39.3%	0.04	549	40.7%	66	40.0%	0.014
Surgical approach, n / %										
Laparoscopic	2244	49.4%	71	42.3%	0.139	594	44.1%	71	43.0%	0.021
Open	1010	22.2%	20	11.9%	0.316	155	11.5%	20	12.1%	0.019
Robotic, including hand assisted	1290	28.4%	77	45.8%	0.344	599	44.4%	74	44.8%	0.008
Indication for surgery, n / %										
Colorectal carcinoma	1410	31.0%	75	44.6%	0.279	603	44.7%	75	45.5%	0.015
Colorectal polyps or polyp syndrome	147	3.2%	3	1.8%	0.108	23	1.7%	3	1.8%	0.009
Diverticulitis	2447	53.9%	53	31.5%	0.491	411	30.5%	52	31.5%	0.022
Inflammatory bowel disease	88	1.9%	8	4.8%	0.134	64	4.7%	7	4.2%	0.023
Other	452	9.9%	29	17.3%	0.196	247	18.4%	28	17.0%	0.037
Teaching hospital, n / %	2594	57.1%	162	96.4%	2.342	1,284	95.3%	160	97.0%	0.1
Hospital bed size 400–499 (vs. 500 +), n / %	3457	76.1%	102	60.7%	0.308	878	65.1%	102	61.8%	0.068

*SD* standard deviation, *SMD* absolute standardized mean difference – an SMD < 0.10 is considered indicative of good balance

*To align identification of diabetes and hypertension between the ECP trial cohort and historical cohort, the category of Diabetes includes subjects whose coded medical history was either ‘Diabetes mellitus’ or ‘Type 2 diabetes mellitus’ and the category of Hypertension includes subjects whose coded medical history was either ‘Hypertension’ or ‘Essential hypertension’

Following propensity score matching, there were only 3 patients in the ECP trial cohort for whom no adequate match could be identified in the historical cohort (98.2% retention), resulting in post-match cohort sizes of 165 patients for the ECP trial cohort and 1,348 patients for the historical cohort. All baseline characteristics were well balanced between the matched study cohorts, as indicated by all covariate SMDs having a value < 0.10 (Fig. 1).

The results to the analysis of study outcomes after propensity score matching in the overall sample are shown in Table 2. Rates are expressed as incidence proportions. Relative to the matched historical cohort, patients in the ECP trial cohort had statistically significant lower conversion rates (4.2% vs. 10.2%, *p* = 0.001), lower 30-day inpatient readmission rates (6.1% vs. 10.8%, *p* = 0.019) as well as lower composite rates of a follow-up care provided in either the inpatient setting, the emergency room, or as a non-elective outpatient visits (6.1% vs. 19.5%, *p* < 0.001). The ECP trial cohort also had lower rates of anastomotic leak (1.8% vs. 6.9%, *p* < 0.001), ileus/small bowel obstruction (4.8% vs. 14.7%, *p* < 0.001), infection (1.8% vs. 5.7%, *p* = 0.001), and bleeding (1.8% vs. 9.2%, *p* < 0.001) during the index admission or within 30 days thereafter, as compared with the historical cohort. No statistically significant differences in rates of pelvic abscess (0.6% vs. 0.4%, *p* = 0.799) and stoma creation (20.6% vs. 19.2%, *p* = 0.676) during the index admission or within 30 days thereafter were found between the two cohorts.

**Table 2:** Primary analysis of outcomes* after propensity score matching

	Historical cohort		ECP trial cohort		MID (95% CI, P)	% Difference in Event Rate
N	1348	100.0%	165	100.0%		
Conversion from minimally-invasive to open surgery, n / %	138	10.2%	7	4.2%	− 6.0% (− 9.5%, − 2.5%; P = 0.001)	58.8%
30-day inpatient readmission, n / %	146	10.8%	10	6.1%	− 4.8% (− 8.8%, − 0.8%; P = 0.019)	43.5%
30-day inpatient/emergency/outpatient visit**, n / %	263	19.5%	10	6.1%	− 13.4% (− 17.7%, − 9.2%; P < 0.001)	68.7%
Anastomotic leak, n / %	93	6.9%	3	1.8%	− 5.1% (− 7.5%, − 2.6%; P < 0.001)	73.9%
Pelvic abscess, n / %	6	0.4%	1	0.6%	0.2% (− 1.1%, 1.4%; P = 0.799)	NS
Ileus/bowel obstruction, n / %	198	14.7%	8	4.8%	− 9.8% (− 13.6%, − 6.1%; P < 0.001)	67.3%
Infection, n / %	77	5.7%	3	1.8%	− 3.9% (− 6.3%, − 1.5%; P = 0.001)	68.4%
Bleeding, n / %	124	9.2%	3	1.8%	− 7.4% (− 9.9%, − 4.8%; P < 0.001)	80.4%
Ostomy, n / %	259	19.2%	34	20.6%	1.4% (− 5.1%, 7.9%; P = 0.676)	NS

*CI* confidence interval, *MID* mean incremental difference (ECP trial cohort minus historical cohort), *NS* not statistically significant

*In the historical cohort, complications were measured using ICD-10-CM/PCS codes from the index admission, or during inpatient, outpatient, or emergency room visits occurring within 30 days of index admission at the same hospital; In the ECP trial, complications were measured during multiple visits including the surgery and postoperative recovery through discharge visit, the follow-up visit at 28 ± 14 days after the procedure date, or during any unplanned visits occurring between

**Composite of inpatient readmission, emergency room visit, or unplanned outpatient visit

### US sub-analyses

**Fig. 2:**
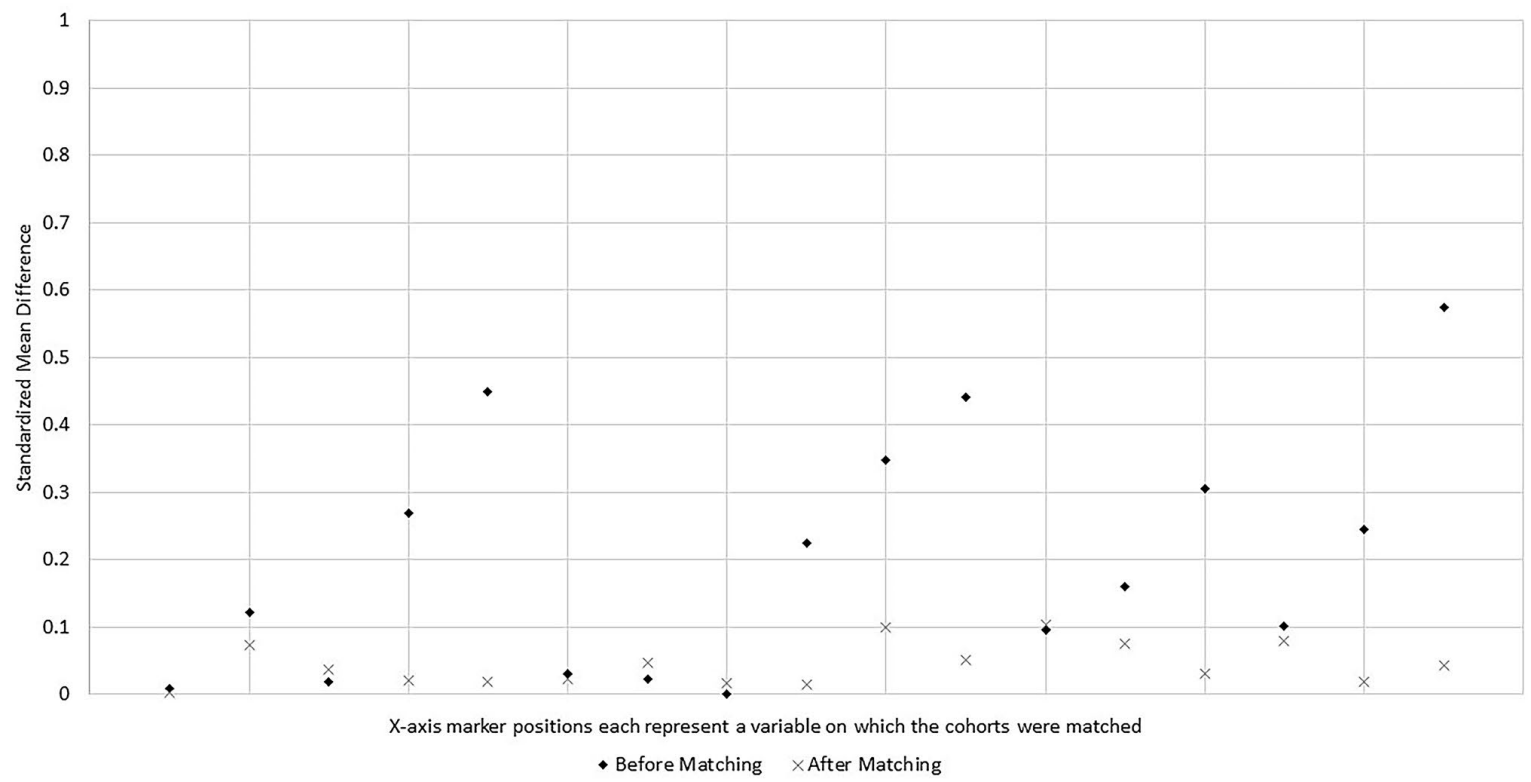
US Sub-analysis analysis: Standardized mean differences before and after matching*. *A standardized mean difference <0.10 is considered indicative of good covariate balance

Baseline characteristics of the study cohorts before and after propensity score matching for the US sub-analysis are shown in Table 3. As all US patients in the ECP trial cohort underwent surgery in teaching hospitals, the pre-matching historical cohort was restricted to patients who underwent surgery in teaching hospitals. A total of 2594 patients met the eligibility criteria for inclusion in the historical cohort, and there were 132 US patients in the ECP trial cohort. Before propensity score matching, the two groups were imbalanced (as indicated by an SMD value ≥ 0.10) with respect to most of the same variables as in the overall study sample (Fig. 2). After propensity score matching, all baseline characteristics were well balanced between the two study cohorts (Fig. 2).

**Table 3:** US Sub-analysis: Cohort characteristics before and after propensity score matching

	Before propensity score matching					After propensity score matching				
	Historical Cohort		ECP trial cohort		SMD	Historical Cohort		ECP trial cohort		SMD
N	2594	100.0%	132	100.0%		947	100.0%	129	100.0%	
Age, mean / SD	59.5	13.0	59.4	12.9	0.01	59.6	13.4	59.6	12.7	0.00
Female, n / %	1436	55.4%	65	49.2%	0.12	490	51.8%	62	48.1%	0.07
Hispanic ethnicity, n / %										
Hispanic	149	5.7%	7	5.3%	0.02	44	4.6%	7	5.4%	0.04
Not Hispanic or Latino	2212	85.3%	122	92.4%	0.27	879	92.8%	119	92.2%	0.02
Not reported	233	9.0%	3	2.3%	0.45	25	2.6%	3	2.3%	0.02
Diabetes*, n / %	401	15.5%	22	16.7%	0.03	170	17.9%	22	17.1%	0.02
Hypertension*, n / %	1247	48.1%	65	49.2%	0.02	455	48.0%	65	50.4%	0.05
Medicare insurance (vs. other)	964	37.2%	49	37.1%	0.00	360	38.0%	48	37.2%	0.02
Surgical approach, n / %										
Laparoscopic	1119	43.1%	43	32.6%	0.23	309	32.6%	43	33.3%	0.02
Open	610	23.5%	16	12.1%	0.35	148	15.7%	16	12.4%	0.10
Robotic, including hand assisted	865	33.3%	73	55.3%	0.44	490	51.7%	70	54.3%	0.05
Indication for surgery, n / %										
Colorectal carcinoma	843	32.5%	49	37.1%	0.10	312	33.0%	49	38.0%	0.10
Colorectal polyps or polyp syndrome	90	3.5%	2	1.5%	0.16	24	2.5%	2	1.6%	0.08
Diverticulitis	1325	51.1%	48	36.4%	0.31	367	38.7%	48	37.2%	0.03
Inflammatory bowel disease	79	3.0%	7	5.3%	0.10	46	4.9%	4	3.1%	0.08
Other	257	9.9%	26	19.7%	0.25	198	20.9%	26	20.2%	0.02
Teaching hospital, n / %	2594	100.0%	132	100.0%	0.00	947	100.0%	129	100.0%	0.00
Hospital bed size 400–499 (vs. 500 +), n / %	2061	79.5%	67	50.8%	0.57	471	49.7%	67	51.9%	0.04

*SD* standard deviation, *SMD* absolute standardized mean difference – an SMD < 0.10 is considered indicative of good balance

*To align identification of diabetes and hypertension between the ECP trial cohort and historical cohort, the category of Diabetes includes subjects whose coded medical history was either ‘Diabetes mellitus’ or ‘Type 2 diabetes mellitus’ and the category of Hypertension includes subjects whose coded medical history was either ‘Hypertension’ or ‘Essential hypertension’

The results of the analysis of study outcomes after propensity score matching within the US sub-analysis, which were congruent with the overall analysis, are shown in Table 4. Rates are expressed as incidence proportions. Relative to the matched historical cohort, patients in the ECP trial cohort had statistically significant lower conversion rates (3.9% vs. 9.9%, P = 0.002), lower 30-day inpatient readmission rates (4.7% vs. 10.0%, P = 0.010) as well as composite rates of a follow-up care provided in either the inpatient setting, the emergency room, or as a non-elective outpatient visits (4.7% vs. 18.2%, P < 0.001). The ECP trial cohort also had lower rates of anastomotic leak (2.3% vs. 6.4%, P = 0.008), ileus/small bowel obstruction (3.1% vs. 14.8%, P < 0.001), infection (0.0% vs. 5.5%, P = 0.002), and bleeding (0.8% vs. 9.7%, P < 0.001) during the index admission or within 30 days thereafter, as compared with the historical cohort. No statistically significant differences in rates of pelvic abscess (0.0% vs. 0.4%, P = 1.000), stoma creation (18.6% vs. 17.8%, P = 0.835), or mean LOS (5.2 days vs. 4.6 days, *p* = 0.245) during the index admission or within 30 days thereafter were found between the two cohorts.

**Table 4:** US Sub-analysis: Analysis of outcomes* after propensity score matching

	Historical Cohort		ECP trial cohort		MID (95% CI, P)**	% Difference in Event Rate
N	947	100.0%	129	100.0%		
Conversion from minimally-invasive to open surgery, n / %	94	9.9%	5	3.9%	− 6.1% (− 9.9%, − 2.2%; P = 0.002)	60.6%
30-day inpatient readmission, n / %	95	10.0%	6	4.7%	− 5.4% (− 9.5%, − 1.3%; P = 0.010)	53.0%
30-day inpatient/emergency/outpatient visit***, n / %	172	18.2%	6	4.7%	− 13.5% (− 17.9%, − 9.1%; P < 0.001)	74.2%
Anastomotic leak, n / %	61	6.4%	3	2.3%	− 4.1% (− 7.2%, − 1.1%; P = 0.008)	64.1%
Pelvic abscess, n / %	4	0.4%	0	0.0%	− 0.4% (P = 1.000)	NS
Ileus/bowel obstruction, n / %	140	14.8%	4	3.1%	− 11.7% (− 15.4%, − 7.9%; P < 0.001)	79.1%
Infection, n / %	52	5.5%	0	0.0%	− 5.5% (P = 0.002)	100.0%
Bleeding, n / %	92	9.7%	1	0.8%	− 8.9% (− 11.4%, − 6.5%; P < 0.001)	91.8%
Ostomy, n / %	169	17.8%	24	18.6%	0.8% (− 6.4%, 7.9%; P = 0.835)	NS
Hospital length of stay for index admission, mean / SD	4.6	4.0	5.2	3.0	0.6 (− 0.4%, 1.6%; P = 0.245)	NS
Median	4.0		3.0			

*CI* confidence interval, *MID* mean incremental difference (ECP trial cohort minus historical cohort), *NS* not statistically significant

*In the historical cohort, complications were measured using ICD-10-CM/PCS codes from the index admission, or during inpatient, outpatient, or emergency room visits occurring within 30 days of index admission at the same hospital; In the ECP trial, complications were measured during multiple visits including the surgery and postoperative recovery through discharge visit, the follow-up visit at 28 ± 14 days after the procedure date, or during any unplanned visits occurring between

**95% confidence intervals and generalized linear model test statistics were not estimable for pelvic abscess and infection due to 0 events in the ECP trial cohort; P-value reported for these two outcomes is based on Fisher’s exact test

***Composite of inpatient readmission, emergency room visit, or unplanned outpatient visit

### Post-hoc Sensitivity Analysis Involving Conversions

The results to the post-hoc sensitivity analysis adjusting for the difference in conversions between the ECP trial cohort and historical cohort are shown in Tables 5 (overall cohorts) and Table 6 (US sub-analysis). In both the overall cohort and the US sub-analysis cohort, the results were nearly identical when adjusting for conversions, with absolute changes in adjusted mean incremental differences never exceeding 0.2%.

**Table 5:** Primary analysis of outcomes* after propensity score matching, when adjusting for conversions as a covariate in outcome models

	Historical Cohort		ECP trial cohort		MID (95% CI, P)**
N	1348	100.0%	165	100.0%	
30-day inpatient readmission, n / %	146	10.8%	10	6.1%	− 4.8% (− 8.8%, − 0.7%; P = 0.020)
30-day inpatient/emergency/outpatient visit***, n / %	263	19.5%	10	6.1%	− 13.4% (− 17.7%, − 9.2%; P < 0.001)
Anastomotic leak, n / %	93	6.9%	3	1.8%	− 5.0% (− 7.5%, − 2.5%; P < 0.001)
Pelvic abscess, n / %	6	0.4%	1	0.6%	0.1% (− 1.2%, 1.4%; P = 0.836)
Ileus/bowel obstruction, n / %	198	14.7%	8	4.8%	− 9.6% (− 13.5%, − 5.7%; P < 0.001)
Infection, n / %	77	5.7%	3	1.8%	− 3.8% (− 6.3%, − 1.4%; P = 0.002)
Bleeding, n / %	124	9.2%	3	1.8%	− 7.3% (− 9.9%, − 4.7%; P < 0.001)
Ostomy, n / %	259	19.2%	34	20.6%	1.5% (− 5.0%, 8.1%; P = 0.646)

*CI* confidence interval, *MID* mean incremental difference (ECP trial cohort minus historical cohort), *NS* not statistically significant

*In the historical cohort, complications were measured using ICD-10-CM/PCS codes from the index admission, or during inpatient, outpatient, or emergency room visits occurring within 30 days of index admission at the same hospital; In the ECP trial, complications were measured during multiple visits including the surgery and postoperative recovery through discharge visit, the follow-up visit at 28 ± 14 days after the procedure date, or during any unplanned visits occurring between

**Adjusting for conversions

***Composite of inpatient readmission, emergency room visit, or unplanned outpatient visit

**Table 6:** US Sub-analysis: Analysis of outcomes* after propensity score matching, when adjusting for conversions as a covariate in outcome models

	Historical Cohort		ECP trial cohort		MID (95% CI, P)**
N	947	100.0%	129	100.0%	
30-day inpatient readmission, n / %	95	10.0%	6	4.7%	− 5.4% (− 9.5%, − 1.3%; *p* = 0.010)
30-day inpatient/emergency/outpatient visit***, n / %	172	18.2%	6	4.7%	− 13.5% (− 17.9%, − 9.1%; *p* < 0.001)
Anastomotic leak, n / %	61	6.4%	3	2.3%	− 4.1% (− 7.1%, − 1.0%; *p* = 0.010)
Pelvic abscess, n / %	4	0.4%	0	0.0%	− 0.4% (*p* = 1.000)
Ileus/bowel obstruction, n / %	140	14.8%	4	3.1%	− 11.5% (− 15.3%, − 7.6%; *p* < 0.001)
Infection, n / %	52	5.5%	0	0.0%	− 5.5% (*p* = 0.002)
Bleeding, n / %	92	9.7%	1	0.8%	− 8.9% (− 11.4%, − 6.5%; *p* < 0.001)
Ostomy, n / %	169	17.8%	24	18.6%	1.2% (− 6.0%, 8.5%; *p* = 0.745)
Hospital length of stay for index admission, mean / SD	4.6	4.0	5.2	3.0	0.8 (− 0.3, 1.8; *p* = 0.159)
Median	4.0		3.0		

*CI* confidence interval, *MID* mean incremental difference (ECP trial cohort minus historical cohort), *NS* not statistically significant

*In the historical cohort, complications were measured using ICD-10-CM/PCS codes from the index admission, or during inpatient, outpatient, or emergency room visits occurring within 30 days of index admission at the same hospital; In the ECP trial, complications were measured during multiple visits including the surgery and postoperative recovery through discharge visit, the follow-up visit at 28 ± 14 days after the procedure date, or during any unplanned visits occurring between

**Adjusting for conversions; 95% confidence intervals and generalized linear model test statistics were not estimable for pelvic abscess and infection due to 0 events in the ECP trial cohort; P-value reported for these two outcomes is based on Fisher’s exact test and therefore remain unadjusted

***Composite of inpatient readmission, emergency room visit, or unplanned outpatient visit

The results to the post-hoc sensitivity analysis excluding patients who were converted to open surgery from the historical cohort and but not excluding them from the ECP cohort are shown in Table 7. The results of this analysis were consistent with those of the primary analyses, with the 30-day inpatient readmission outcome becoming statistically insignificant (P = 0.056) and the general magnitudes of the mean incremental differences being slightly attenuated (e.g., in the primary analyses, the mean incremental differences for anastomotic leak was -5.1% vs. -4.8% in this sensitivity analysis).

**Table 7:** Primary analysis of outcomes* after propensity score matching, when excluding conversions from the historical cohort

	Historical Cohort		ECP trial cohort		MID (95% CI, *p*)**
N	1210	100.0%	165	100.0%	
30-day inpatient readmission, n / %	134	11.1%	10	6.1%	− 5.0% (− 10.1%, 0.1%; *p* = 0.056)
30-day inpatient/emergency/outpatient visit***, n / %	236	19.5%	10	6.1%	− 13.4% (− 19.4%, − 7.4%; *p* = 0.000)
Anastomotic leak, n / %	80	6.6%	3	1.8%	− 4.8% (− 7.1%, − 2.5%; *p* = 0.000)
Pelvic abscess, n / %	6	0.5%	1	0.6%	0.1% (− 1.2%, 1.4%; *p* = 0.839)
Ileus/bowel obstruction, n / %	165	13.6%	8	4.8%	− 8.8% (− 13.5%, − 5.4%; *p* = 0.000)
Infection, n / %	66	5.4%	3	1.9%	− 3.6% (− 6.2%, − 0.9%; *p* = 0.009)
Bleeding, n / %	118	9.8%	3	1.8%	− 8.0% (− 11.4%, − 4.6%; *p* = 0.000)
Ostomy, n / %	228	18.8%	35	20.9%	2.1% (− 7.5%, 11.7%; *p* = 0.665)

*CI* confidence interval, *MID* mean incremental difference (ECP trial cohort minus historical cohort), *NS* not statistically significant

*In the historical cohort, complications were measured using ICD-10-CM/PCS codes from the index admission, or during inpatient, outpatient, or emergency room visits occurring within 30 days of index admission at the same hospital; In the ECP trial, complications were measured during multiple visits including the surgery and postoperative recovery through discharge visit, the follow-up visit at 28 ± 14 days after the procedure date, or during any unplanned visits occurring between

**When excluding conversions from the historical cohort, applying cluster-robust standard errors, and adjusting for slight imbalance in teaching status and hospital bed size

***Composite of inpatient readmission, emergency room visit, or unplanned outpatient visit

The results to the post-hoc sensitivity analysis stratifying the historical cohort into tertiles with respect to their institution-level conversion rate based on the patients included in the historical cohort are shown in Table 8. The results of this analysis were also largely consistent with those of the primary analyses. For example, in the primary analyses, the mean incremental differences for anastomotic leak were -5.1% vs. -4.9%, -4.1%, and -7.2% in the bottom, middle, and top tertiles of historical cohort. Findings were most attenuated in the bottom tertile for 30-day inpatient readmission, bleeding, and ostomy. Findings were most attenuated in the middle tertile for anastomotic leak, ileus/bowel obstruction, and infection. The 30-day inpatient readmission (bottom and top tertiles) and infection (middle tertile) outcomes lost statistical significance.

**Table 8:** Primary analysis of outcomes* after propensity score matching, when stratifying historical cohort by tertiles of institution-specific conversion rates

	Historical cohort		ECP trial cohort		MID	95% CI		p**
	1348	100.0%	165	100.0%				
30-day inpatient readmission, adjusted %								
Bottom tertile	9.6%		6.1%		3.5%	− 2.3%	9.4%	0.240
Middle tertile	12.1%				6.0%	0.2%	11.9%	0.043
Top tertile	11.6%				5.5%	− 0.3%	11.4%	0.063
30-day inpatient/emergency/outpatient visit***, adjusted %								
Bottom tertile	21.0%		6.1%		14.9%	5.9%	23.8%	0.001
Middle tertile	19.2%				13.1%	6.9%	19.3%	0.000
Top tertile	18.1%				12.0%	5.6%	18.5%	0.000
Anastomotic leak, adjusted %								
Bottom tertile	6.8%		1.8%		4.9%	2.4%	7.5%	0.000
Middle tertile	5.9%				4.1%	1.1%	7.0%	0.007
Top tertile	9.0%				7.2%	3.8%	10.6%	0.000
Pelvic abscess, adjusted %								
Bottom tertile	Model failed to converge due to infrequency of outcome across stratifications							
Middle tertile								
Top tertile								
Ileus/bowel obstruction, adjusted %								
Bottom tertile	14.2%		3.4%		10.9%	5.9%	15.8%	0.000
Middle tertile	11.9%				8.5%	3.2%	13.9%	0.002
Top tertile	19.1%				15.7%	11.4%	20.0%	0.000
Infection, adjusted %								
Bottom tertile	5.9%		1.9%		4.0%	0.9%	7.2%	0.013
Middle tertile	4.5%				2.6%	− 1.0%	6.2%	0.158
Top tertile	6.3%				4.5%	1.3%	7.7%	0.006
Bleeding, adjusted %								
Bottom tertile	5.6%		1.9%		3.8%	0.3%	7.3%	0.035
Middle tertile	10.0%				8.2%	4.5%	11.8%	0.000
Top tertile	15.0%				13.1%	6.8%	19.4%	0.000
Ostomy, adjusted %								
Bottom tertile	16.3%		21.6%		− 5.3%	− 13.9%	3.4%	0.233
Middle tertile	21.6%				0.0%	− 8.4%	8.5%	0.999
Top tertile	19.6%				− 2.0%	− 9.8%	5.9%	0.622

*CI* confidence interval, *MID* mean incremental difference (ECP trial cohort minus historical cohort), *NS* not statistically significant

*In the historical cohort, complications were measured using ICD-10-CM/PCS codes from the index admission, or during inpatient, outpatient, or emergency room visits occurring within 30 days of index admission at the same hospital; In the ECP trial, complications were measured during multiple visits including the surgery and postoperative recovery through discharge visit, the follow-up visit at 28 ± 14 days after the procedure date, or during any unplanned visits occurring between

**When adjusting for all matching covariates and applying cluster-robust standard errors; marginal standardization was used to generate adjusted outcome rates and mean incremental differences with P-values

***Composite of inpatient readmission, emergency room visit, or unplanned outpatient visit

## Discussion

This study assessed outcomes of the ECP trial cohort as indirectly compared with a retrospectively established matched historical cohort of patients undergoing left-sided colorectal reconstructions with manual circular staplers. Using this matching-adjusted indirect comparison framework, the ECP was associated with significantly lower rates (incidence proportions) of several peri- and post-operative complications as well as 30-day readmissions.

With respect to complications, the ECP trial cohort had statistically significant lower rates of anastomotic leaks, ileus/bowel obstruction, infection, and bleeding as compared with the matched historical cohort. The surgical complications examined within this study, particularly anastomotic leak, infection, and bleeding, have been shown to be clinically impactful both in the short and long-term and have substantial economic ramifications for hospitals and payers, including increases in hospital- and payer-borne costs, LOS, risks of discharge to institutional post-acute care, the risks of hospital readmissions [14].

Though striking, the relative incidence of these complications must be interpreted in the context of the MAIC approach and use of different methods of complication ascertainment between the ECP trial cohort and historical cohort. Specifically, whereas complications were prospectively identified through case report forms in the ECP trial, they were retrospectively identified through ICD-10-CM/PCS coding in the historical cohort (see Supplemental Appendix 1). The impact of differing methods of ascertainment was likely ambiguous: within the historical cohort, only those complications that are deemed severe enough to warrant coding are likely to be documented in a medical record, and thus identification of surgical complications via ICD codes may associated with high positive predictive value, but low sensitivity; in contrast, active concerted prospective ascertainment of complications may have led to greater sensitivity. Use of ICD-10-CM/PCS coding may also result in false positives; however, to reduce the risk of false positives we required that all complication diagnoses not be designated as ‘present on admission,’ which is an administrative designation made within hospital records to delineate those conditions that were pre-existing upon admission vs. those that developed during the admission. Nevertheless, some code descriptions, such as those associated with anastomotic leak, are non-specific. Similarly, the ICD-10-PCS codes for bleeding are relatively nonspecific. Circular staplers are most likely to affect anastomotic bleeding. In the historical cohort, the most frequently-observed ICD-10-CM codes related to bleeding were “Postprocedural hemorrhage of a digestive system organ or structure following a digestive system procedure” and “Melena.” Notably, anastomotic bleeding would indeed be coded to “Postprocedural hemorrhage of a digestive system organ or structure following a digestive system procedure” and melena is a common way in which anastomotic bleeding may present. As data on ECP begin to accrue in real-world databases such as the PHD, future analyses directly and concurrently comparing ECP with manual circular staplers using the same measurement mechanisms will be possible.

Although each endpoint was examined separately, they are closely related with one another. Anastomotic leak, infection, and ileus/bowel obstruction tend to co-present. Indeed, of the 96 patients who had a documented anastomotic leak 58 (60.4%) had a documented ileus/bowel obstruction, 45 (46.9%) had a documented infection, 43 (44.8%) had a 30-day inpatient readmission, and 52 (54.2%) had 30-day encounter of follow-up care in either the inpatient setting, the emergency room, or as a non-elective outpatient visit. In the historical cohort, it is also possible that an anastomotic leak may have been coded as an infection. When considering leak and infection as a composite, the difference between the ECP cohort and historical cohort remained pronounced (9.3% vs. 3.6%, respectively). Ultimately, the findings of the present study must not be interpreted as being additive across surgical complications, but rather understood in the context of their close relationships with one another.

Notably, the present study’s findings related to anastomotic leak are consistent with those reported by Pla-Marti and colleagues [5]. They reported that among patients undergoing left-sided colorectal anastomosis, anastomotic leak was observed in 11.8% of 218 patients (5.8% requiring reoperation) in whom manual circular staplers were used and 1.7% of 61 patients in whom the ECP was used (P = 0.022). Furthermore, because that study was conducted within a single institution using consistent methods of outcome ascertainment, it provides important confirmatory results that address some of the limitations of the present study.

Other previous studies using direct, as opposed to indirect, comparisons within the same database or institution have reported an association of powered firing and/or Gripping Surface Technology (both of which are incorporated into the ECP) with selected benefits for non-circular surgical staplers (the most pronounced and consistent of which has been lower risks of hemorrhagic complications), including: Roy et al. [15] for patients undergoing laparoscopic bariatric surgery; Miller et al. [16] for patients undergoing video-assisted thoracoscopic lobectomy of the lung; Park et al. [17] patients undergoing thoracoscopic lobectomy of the lung; and Rawlins et al. [18] for undergoing sleeve gastrectomy.

Two additional findings related to anastomotic leak in the present study warranted further investigation after examination of study results. First, after matching, 165 of 168 patients were retained in the ECP trial cohort. Upon review of the matched ECP trial cohort outcomes, 3 of the 165 patients in the ECP trial had anastomotic leak, as compared with 4 of the 168 patients reported by Herzig et al.^5^ Under the hypothetical situation that the single patient with anastomotic leak who was not matched during the matching procedure had been retained in the sample, the findings related to anastomotic leak would have changed minimally (2.4% ECP trial cohort vs. 6.9% historical cohort, mean incremental difference = -4.5%; 95% CI = -72%, 1.8%; P = 0.001). Second, of the 93 patients who experienced anastomotic leak in the historical control cohort, 40 (43.0%) received the initial diagnosis after discharge from the index admission. This might partially explain the why differences in leak were statistically significant but differences in LOS were not: anastomotic leaks occurring after discharge would have no influence over the index admission’s LOS.

The ECP trial cohort experienced a statistically significant lower rate of conversion from minimally invasive to open surgery as compared with the historical cohort. As conversions are often undertaken prior to performing the circular anastomosis, the absence of this event may be a surrogate for expertise with minimally invasive procedures, experience of surgeons, and differences in the difficulties of procedures. Therefore, we conducted several post-hoc sensitivity analyses wherein all study outcomes were reanalyzed when adjusting for the difference in conversions between the ECP trial cohort and the historical cohort, when excluding patients with conversions from the historical cohort only, and when stratifying the historical cohort by institution-specific conversion rates. The results from these post-hoc sensitivity analysis were consistent with those of the main analyses, suggesting that the differing conversion rates did not confound the association of the study groups with the study outcomes.

### Limitations

This analysis is subject to additional limitations. First, this matching adjusted indirect comparison was a retrospective study without randomization. Although we used propensity score matching to balance the two cohorts on many important prognostic factors, there were many other factors that we were unable to match on due to lack of data, such as surgeon skill level, experience (e.g., staff vs. trainee), and case volume, certain surgical aspects of the procedure (e.g. end-to-end vs. side-to-side anastomosis, level/distance of anastomosis from the anal verge), and other patient and practice factors (e.g. neoadjuvant treatments, mechanical bowel preparation, use of indocyanine green fluorescence imaging, or other adjuncts).

The inability to account for such factors would affect the study results primarily if the sites and investigators of the ECP trial differed systematically from those in the historical cohort after matching. Trial sites and investigators are often chosen specifically to be high-quality and highly experienced, which would likely not have been fully accounted for by matching on number of hospital beds and teaching vs. non-teaching. Patients participating in clinical trials may also receive generally better care and careful follow-up as compared with those receiving ‘standard’ care. Some of this potential confounding may have been counterbalanced by the fact that the ECP trial represented the investigator’s first experience with the ECP in clinical practice. Furthermore, as noted above, the single-institution analysis conducted by Pla-Marti and colleagues^5^ yielded findings on anastomotic leak risk that were similar to those reported in the present study.

Second, there were outcomes that we were unable to measure from the PHD, and therefore could not compare between the study groups, including technical failure of the stapler and ergonomics of the stapler firing, among others. As these measures are unavailable in large real-world databases, prospective studies would be needed to assess them.

Finally, the ECP trial cohort was relatively small and came from both the US and Europe. Future larger retrospective studies would help to verify the generalizability of the study findings. In the US sub-analyses, findings were consistent in direction with the overall analysis.

## Conclusion

In this propensity score matching-adjusted indirect comparison of patients undergoing left-sided colorectal reconstructions, the ECP trial cohort had lower risks of several surgical complications and 30-day readmissions as compared with a retrospectively established historical cohort for whom manual circular staplers were used. Further controlled prospective clinical studies are needed to confirm the validity of this finding.

## Supplementary Information

Below is the link to the electronic supplementary material.

Supplementary file1 (DOCX 22 KB)
