# Supplementary material for: Outcomes associated with the use of a new powered circular stapler for left-sided colorectal reconstructions: a propensity score matching-adjusted indirect comparison with manual circular staplers
Source: Surg Endosc. 2021 May 24;36(4):2541–53. doi: 10.1007/s00464-021-08542-7 (PMC8921098; doi:10.1007/s00464-021-08542-7)
Supplement: Supplementary file 1 — Supplementary file1 (DOCX 22 KB) [file 464_2021_8542_MOESM1_ESM.docx]

Supplemental Appendix 1. Observed complication codes in historical cohort and corresponding ECP trial adverse event terms

| **ICD Code** | **ICD Code Description** | **Complication** | **# of Patients*** | **ECP trial**  **adverse event terms** |
| --- | --- | --- | --- | --- |
| K91.89 | Other postprocedural complications and disorders of digestive system | Anastomotic leak | 53 | Anastomotic leak |
| K65.1 | Peritoneal abscess | Anastomotic leak | 36 |  |
| K63.2 | Fistula of intestine | Anastomotic leak | 19 |  |
| D62 | Acute posthemorrhagic anemia | Bleeding | 92 | Blood in stool  PR bleeding  Post Op Bleed |
| K91.840 | Postprocedural hemorrhage of a digestive system organ or structure following a digestive system procedure | Bleeding | 13 |  |
| K92.1 | Melena | Bleeding | 13 |  |
| L76.32 | Postprocedural hematoma of skin and subcutaneous tissue following other procedure | Bleeding | 6 |  |
| K91.872 | Postprocedural seroma of a digestive system organ or structure following a digestive system procedure | Bleeding | 5 |  |
| K92.2 | Gastrointestinal hemorrhage, unspecified | Bleeding | 4 |  |
| K91.870 | Postprocedural hematoma of a digestive system organ or structure following a digestive system procedure | Bleeding | 3 |  |
| K91.871 | Postprocedural hematoma of a digestive system organ or structure following other procedure | Bleeding | 1 |  |
| K91.873 | Postprocedural seroma of a digestive system organ or structure following other procedure | Bleeding | 1 |  |
| R58 | Hemorrhage, not elsewhere classified | Bleeding | 1 |  |
| K56.7 | Ileus, unspecified | Ileus/bowel obstruction | 118 | Ileus  Large bowel obstruction  partial obstruction at rectal sheath  Pseudo obstruction due to distended colon  small bowel obstruction  ileostomy obstruction  Ileostomy-obstruction  post op ileus  post-op ileus |
| K56.699 | Other intestinal obstruction unspecified as to partial versus complete obstruction | Ileus/bowel obstruction | 19 |  |
| K56.69 | Other intestinal obstruction | Ileus/bowel obstruction | 16 |  |
| K56.60 | Unspecified intestinal obstruction | Ileus/bowel obstruction | 10 |  |
| K56.0 | PARALYTIC ILEUS | Ileus/bowel obstruction | 6 |  |
| K56.5 | Paralytic ileus and intestinal obstruction without hernia | Ileus/bowel obstruction | 4 |  |
| K91.31 | Postprocedural partial intestinal obstruction | Ileus/bowel obstruction | 3 |  |
| K56.50 | Intestinal adhesions [bands], unspecified as to partial versus complete obstruction | Ileus/bowel obstruction | 3 |  |
| K56.600 | Partial intestinal obstruction, unspecified as to cause | Ileus/bowel obstruction | 3 |  |
| K56.690 | Other partial intestinal obstruction | Ileus/bowel obstruction | 2 |  |
| K50.112 | Crohn's disease of large intestine with intestinal obstruction | Ileus/bowel obstruction | 1 |  |
| K51.512 | Left sided colitis with intestinal obstruction | Ileus/bowel obstruction | 1 |  |
| K51.912 | Ulcerative colitis, unspecified with intestinal obstruction | Ileus/bowel obstruction | 1 |  |
| K91.30 | Postprocedural intestinal obstruction, unspecified as to partial versus complete | Ileus/bowel obstruction | 1 |  |
| K56.51 | Intestinal adhesions [bands], with partial obstruction | Ileus/bowel obstruction | 1 |  |
| K56.52 | Intestinal adhesions [bands] with complete obstruction | Ileus/bowel obstruction | 1 |  |
| K59.8 | Other specified functional intestinal disorders: Pseudo-obstruction (acute) (chronic) of intestine | Ileus/bowel obstruction | 1 |  |
| T81.4XXA | Infection following a procedure, initial encounter | Infection | 44 | bacteremia with fever  wound infection |
| R65.21 | Spontaneous bacterial peritonitis | Infection | 13 |  |
| K65.0 | Generalized (acute) peritonitis | Infection | 3 |  |
| K65.9 | Peritonitis, unspecified | Infection | 3 |  |
| T81.43XA | Infection following a procedure, organ and space surgical site, initial encounter | Infection | 3 |  |
| T81.49XA | Infection following a procedure, other surgical site, initial encounter | Infection | 3 |  |
| T81.41XA | Infection following a procedure, superficial incisional surgical site, initial encounter | Infection | 2 |  |
| R65.20 | Severe sepsis without septic shock | Infection | 1 |  |
| T81.40XA | Infection following a procedure, unspecified, initial encounter | Infection | 1 |  |
| T81.44XA | Sepsis following a procedure, initial encounter | Infection | 1 |  |
| 0D1B4Z4 | Bypass Ileum to Cutaneous, Percutaneous Endoscopic Approach | Ostomy | 121 | Ostomy |
| 0D1B0Z4 | Bypass Ileum to Cutaneous, Open Approach | Ostomy | 115 |  |
| 0D1M0Z4 | Bypass Descending Colon to Cutaneous, Open Approach | Ostomy | 13 |  |
| 0D1L0Z4 | Bypass Transverse Colon to Cutaneous, Open Approach | Ostomy | 5 |  |
| 0D1M4Z4 | Bypass Descending Colon to Cutaneous, Percutaneous Endoscopic Approach | Ostomy | 2 |  |
| 0D1N4Z4 | Bypass Sigmoid Colon to Cutaneous with Autologous Tissue Substitute, Percutaneous Endoscopic Approach | Ostomy | 2 |  |
| 0D1L4Z4 | Bypass Transverse Colon to Cutaneous, Percutaneous Endoscopic Approach | Ostomy | 1 |  |
| 0D1N074 | Bypass Sigmoid Colon to Cutaneous with Autologous Tissue Substitute, Open Approach | Ostomy | 1 |  |
| 0D1N0Z4 | Bypass Sigmoid Colon to Cutaneous, Open Approach | Ostomy | 1 |  |
| K68.11 | Postprocedural retroperitoneal abscess | Pelvic Abscess | 4 | Pelvic abscess |
| K63.0 | Abscess of intestine | Pelvic Abscess | 3 |  |

*The number of patients across categories are not mutually exclusive
